# Supplementary material for: A periplasmic cinched protein is required for siderophore secretion and virulence of Mycobacterium tuberculosis
Source: Nat Commun. 2022 Apr 26;13:2255. doi: 10.1038/s41467-022-29873-6 (PMC9042941; doi:10.1038/s41467-022-29873-6)
Supplement: Supplementary file 1 — Supplementary Information [file 41467_2022_29873_MOESM1_ESM.pdf]

Supplementary Information

A periplasmic cinched protein is required for siderophore secretion and virulence of *Mycobacterium tuberculosis*

Lei Zhang[1], James E. Kent[2], Meredith Whitaker[3], David C. Young[4], Dominik Herrmann[1], Alexander E. Aleshin[2], Ying-Hui Ko[5], Gino Cingolani[5], Jamil S. Saad[1], D. Branch Moody[4], Francesca M. Marassi[2], Sabine Ehrt[3] and Michael Niederweis[1]*

[1] Department of Microbiology, University of Alabama at Birmingham, Birmingham, AL 35294, USA

[2] Cancer Center, Sanford Burnham Prebys Medical Discovery Institute, La Jolla, CA 92037, USA

[3] Department of Microbiology and Immunology, Weill Cornell Medical College, New York, NY 10021, USA

[4] Division of Rheumatology, Inflammation and Immunity, Brigham and Women's Hospital, Harvard Medical School, Boston, MA 02115, USA

[5] Department of Biochemistry and Molecular Biology, Thomas Jefferson University, Philadelphia, PA 19107, USA

*Address correspondence to: Michael Niederweis, mnieder@uab.edu

Supplementary Figures

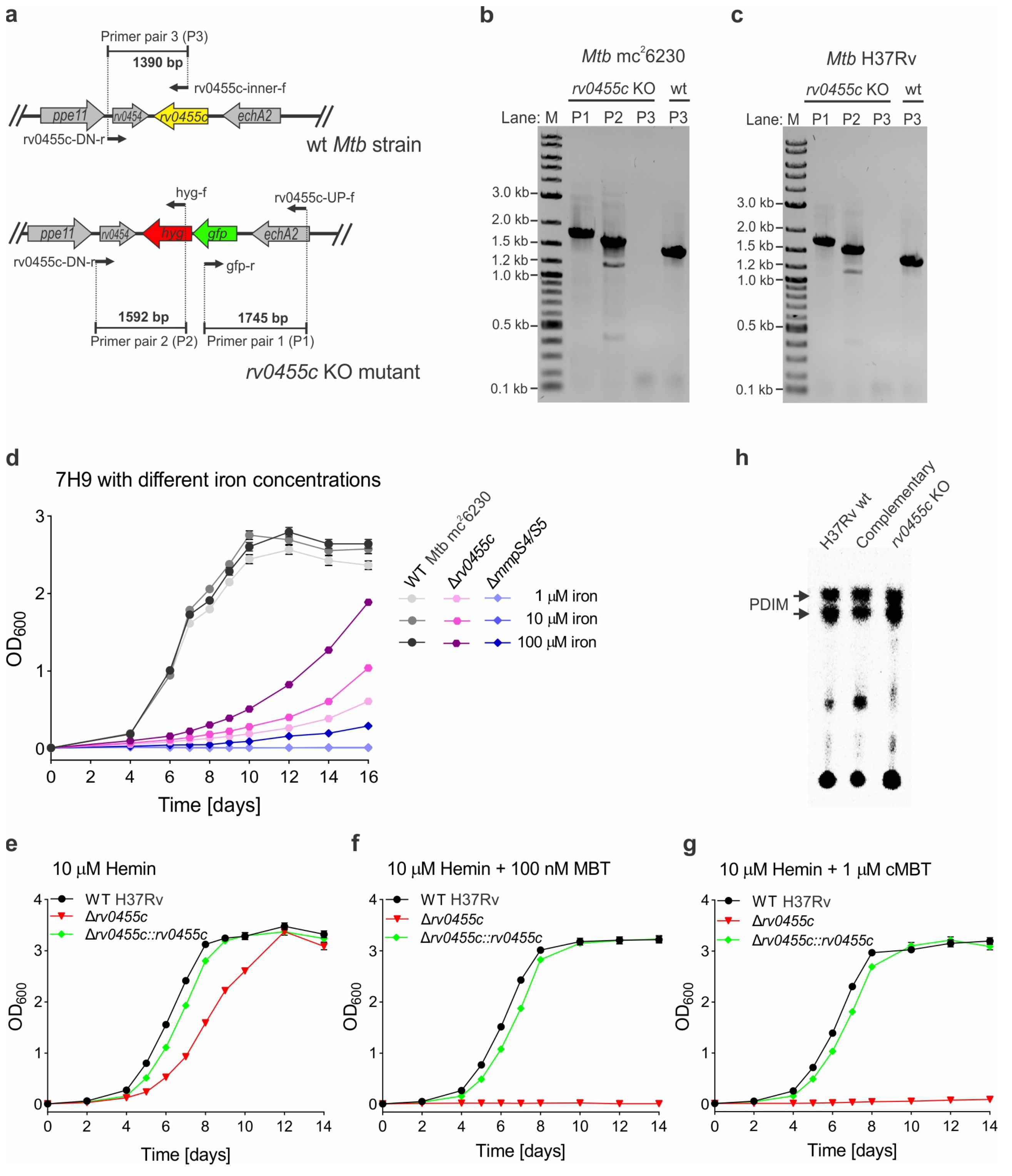

Figure S1. Construction of *M. tuberculosis rv0455c* deletion mutants and growth assays.
a. Schematic representation of the *rv0455c* genomic regions in Mtb H37Rv and PCR performed to validate deletion of *rv0455c*. **b, c.** Validation of the *rv0455c* deletion (KO) mutants (Mtb ML2203 and ML2700) in the Mtb mc^{2}6230 (H37Rv ΔRD1 Δ*panCD*) strain and Mtb H37Rv strain (Table S1) by PCR

using primers (Table S3). **d.** Growth curves of the Mtb mc[2]6230 (wt), the Δ*rv0455c* mutant (ML2203) and the Δ*mmpS4/S5* mutant (ML859) in the 7H9 medium with different Fe^{3+} concentrations (1 µM, 10 µM, 100 µM ferric ammonium citrate). The initial OD_{600} of all cultures was 0.01. Error bars represent standard deviations from the mean results of biological triplicates (n = 3). Source data are provided in the Source Data file. **e-g.** Growth curves of the Mtb H37Rv (wt), the Δ*rv0455c* mutant (ML2700) and the complementation strain (ML2701) in low-iron 7H9/ADS medium supplemented with (**e**) 10 µM hemin, (**f**) 10 µM hemin plus 100 nM Fe-MBT, and (**g**) 10 µM hemin plus 1 µM Fe-cMBT, respectively. The initial OD_{600} of all cultures was 0.012. Error bars represent standard deviations from the mean results of biological triplicates (n = 3). Source data are provided in the Source Data file. **h.** Phthiocerol dimycocerosate analysis. Mtb strains used for virulence studies wt (ML2710), Δ*rv0455c* (ML2711) and the complemented strain ML2701 were labeled with ^{14}C-propionate for two days. Lipids were extracted with petroleum ether and separated by TLC using a mixture of petroleum ether and diethyl ether (9:1) as a solvent. Radioactive lipids were visualized using a Phosphoimager. Data were obtained from at least two independent experiments and representative images are shown.

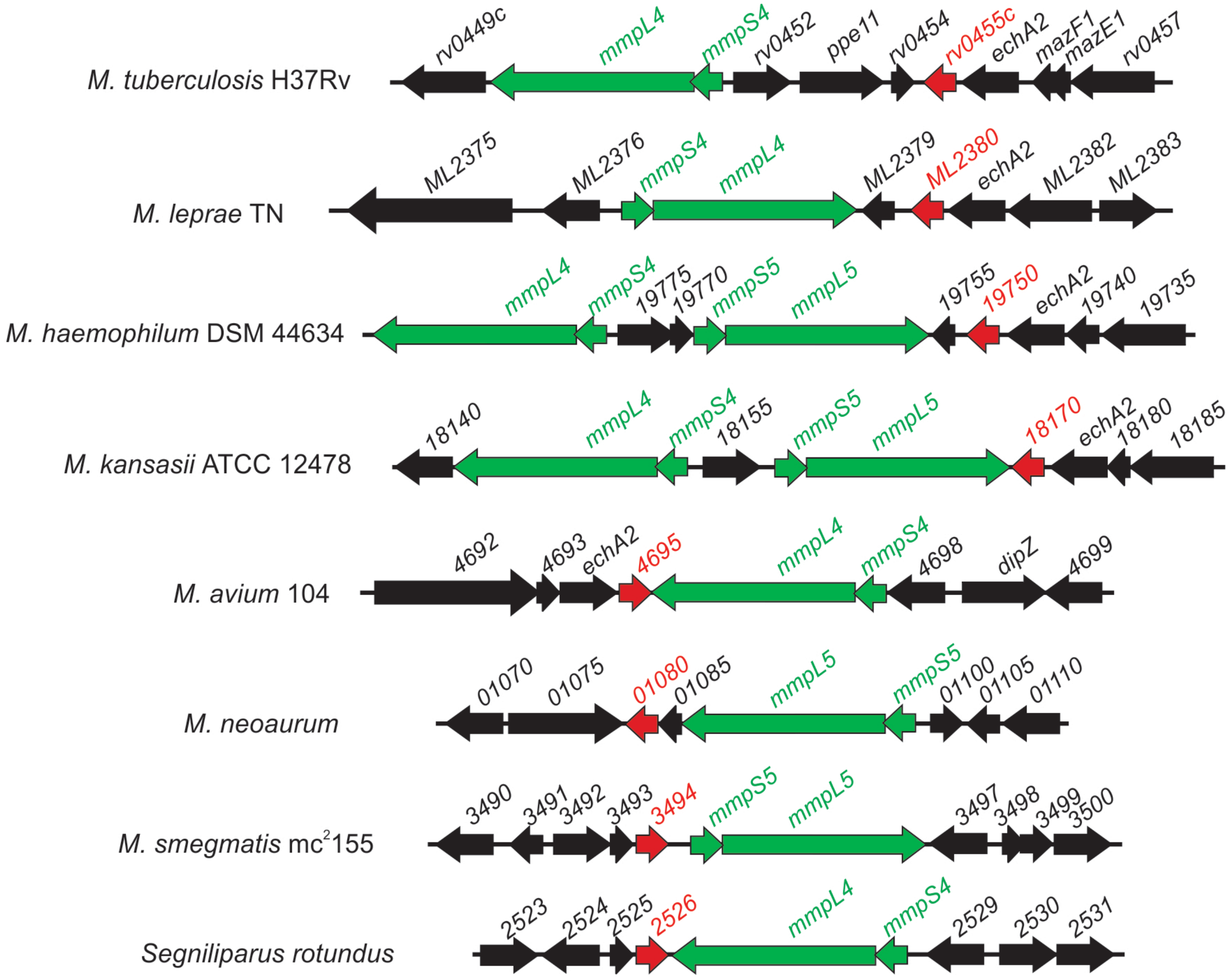

Figure S2. Genomic location of the *rv0455c* gene close to genes encoding the RND family efflux pumps.

The *rv0455c* gene and its homologs (red) and the genes encoding the putative siderophore efflux pumps *mmpS4/L4* (green) or *mmpS5/L5* (green) are shown in mycobacterial species and in the related *Segniliparus rotundus*.

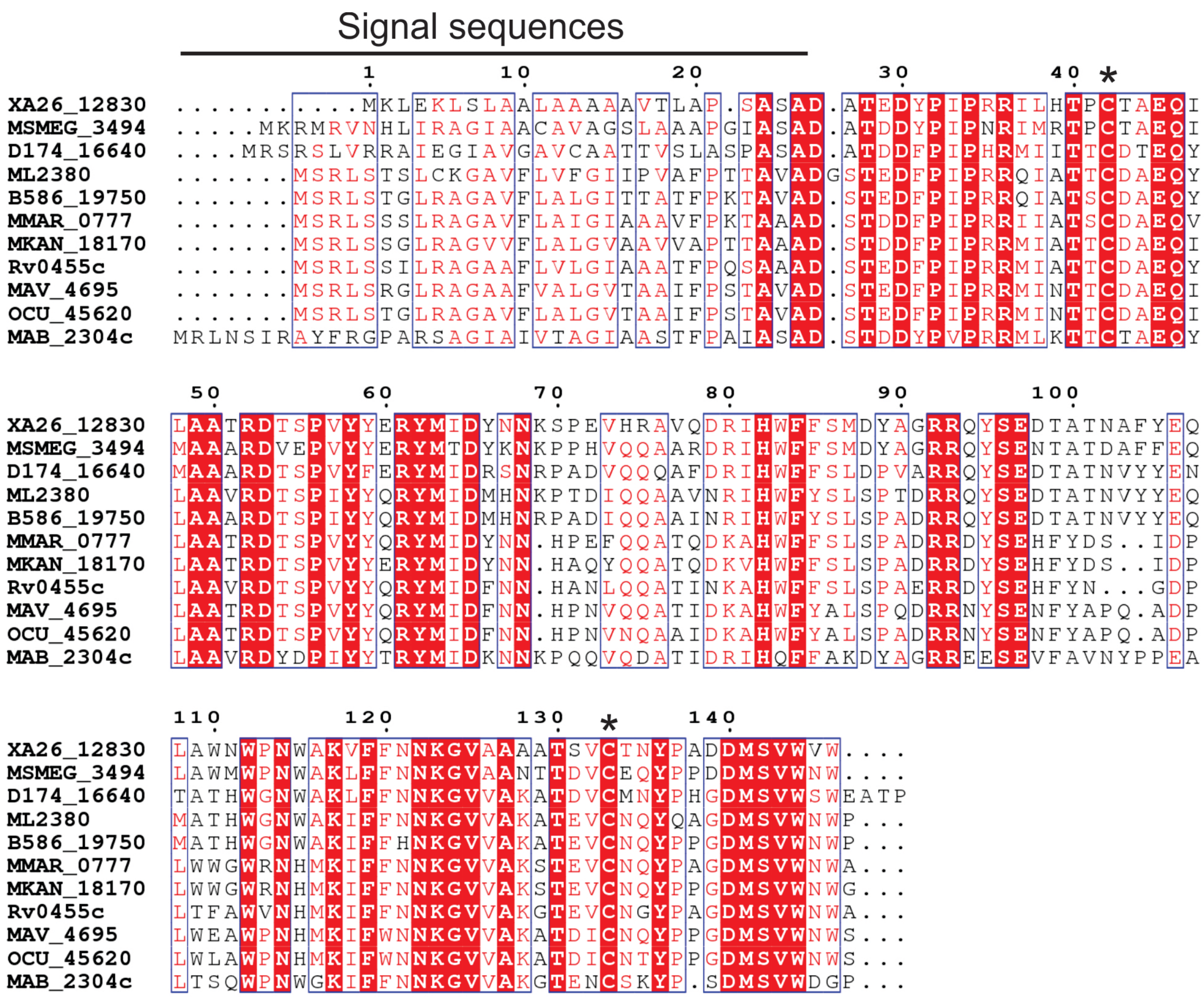

Figure S3. The homologs of Rv0455c share high sequence similarity in mycobacteria.
Protein sequence alignment by ClustalW for the homologs of Rv0455c including MSMEG_3494 of *M. smegmatis* mc^{2}155, ML2380 of *M. leprae* TN, B586_19750 of *M. haemophilum* DSM 44634, MAV_4695 of *M. avium* 104, MKAN_18170 of *M. kansasii* ATCC 12478, MMAR_0777 of *M. marinum* M, MAB_2304c of *M. abscessus* ATCC 19977, XA26_12830 of *M. fortuitum*, D174_16640 of *M. neoaurum*, OCU_45620 of *M. intracellulare* ATCC 13950. Note: MAV_4695 and OCU_45620 are shortened in the N-terminus by 28- and 12-amino acid, respectively, in the current versions in Genbank, probably due to incorrect definition for the length of the ORFs. The intact MAV_4695 and OCU_45620 are shown in this alignment. The two conserved cysteines are marked with asterisks (*).

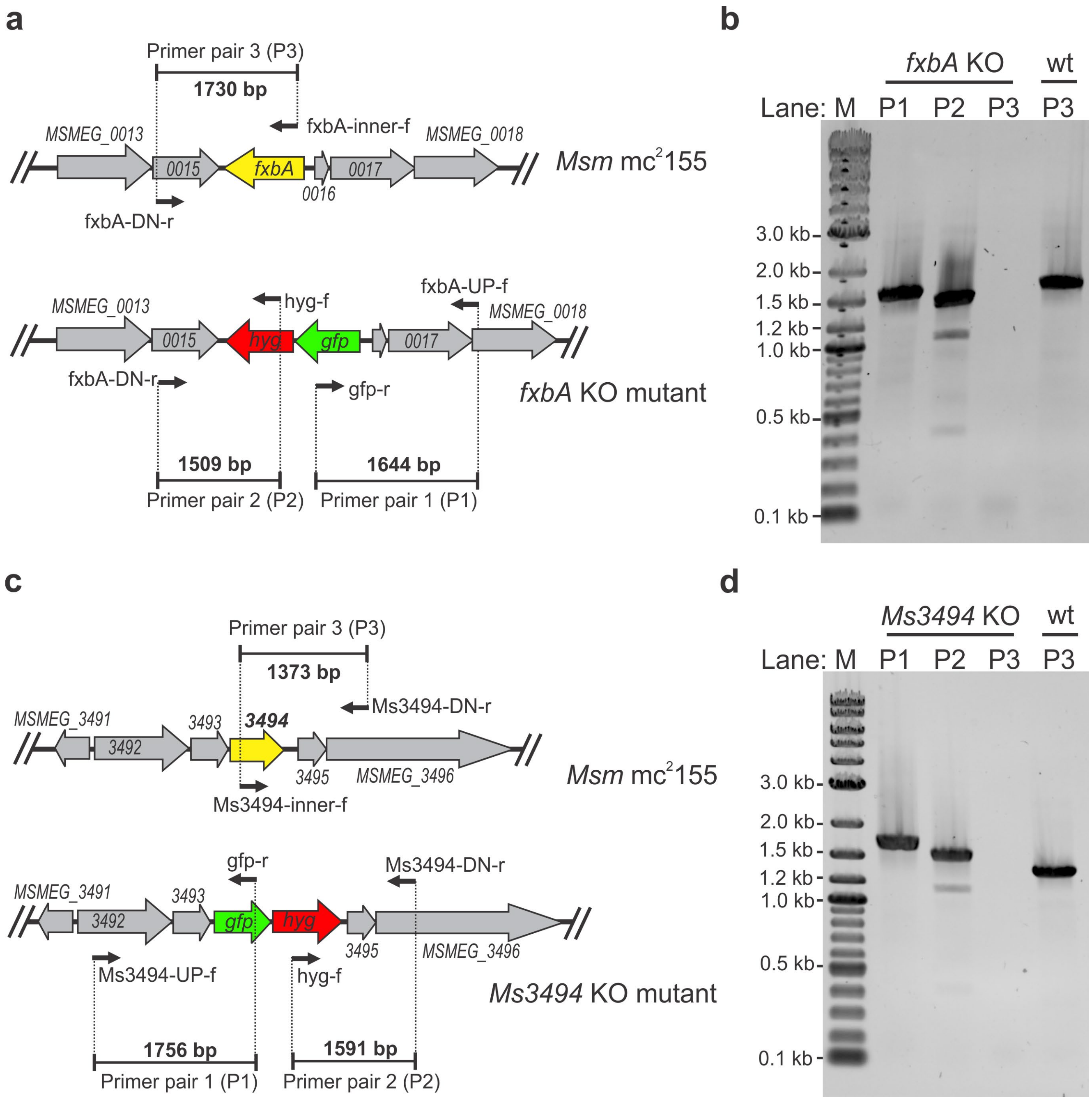

Figure S4. Construction of the *M. smegmatis fxbA* and *msmeg_3494* deletion mutants.

a. Schematic representation of the *fxbA* genomic regions in *M. smegmatis* mc^{2}155 and PCR performed to validate deletion of *fxbA*. **b.** PCR using primers (Table S3) to validate the *fxbA* knock out (KO) (Msm ML2248) in *M. smegmatis* mc^{2}155. **c.** Schematic representation of the *MSMEG_3494* genomic regions in *M. smegmatis* mc^{2}155 and PCR performed to validate deletion of *MSMEG_3494*. **d.** PCR using primers (Table S3) to validate the *MSMEG_3494* knock out (KO) (Msm ML2275) in *M. smegmatis* Δ*fxbA::loxP* strain.

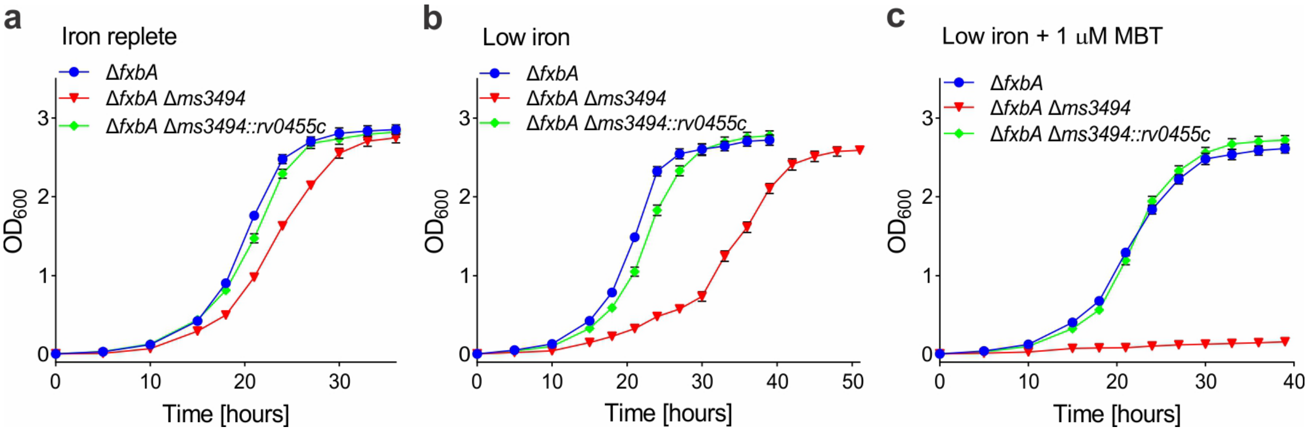

Figure S5. Rv0455c and its *M. smegmatis* homolog Ms3494 have the same function.
Growth curves of *M. smegmatis* strains Δ*fxbA* (ML2249), Δ*fxbA/*Δ*msmeg_3494* (ML2275) and Δ*fxbA/*Δ*msmeg_3494::rv0455c* (ML2276) in self-made low-iron (less than 0.1 μM Fe^{3+}) 7H9 medium supplemented with 150 μM ammonium ferric citrate (**a**), 1 μM ammonium ferric citrate (**b**), and 1 μM ferric-mycobactin (**c**), respectively. The initial OD_{600} of all cultures is 0.005. Error bars represent standard deviations from the mean results of biological triplicates (*n* = 3). Source data are provided in the Source Data file.

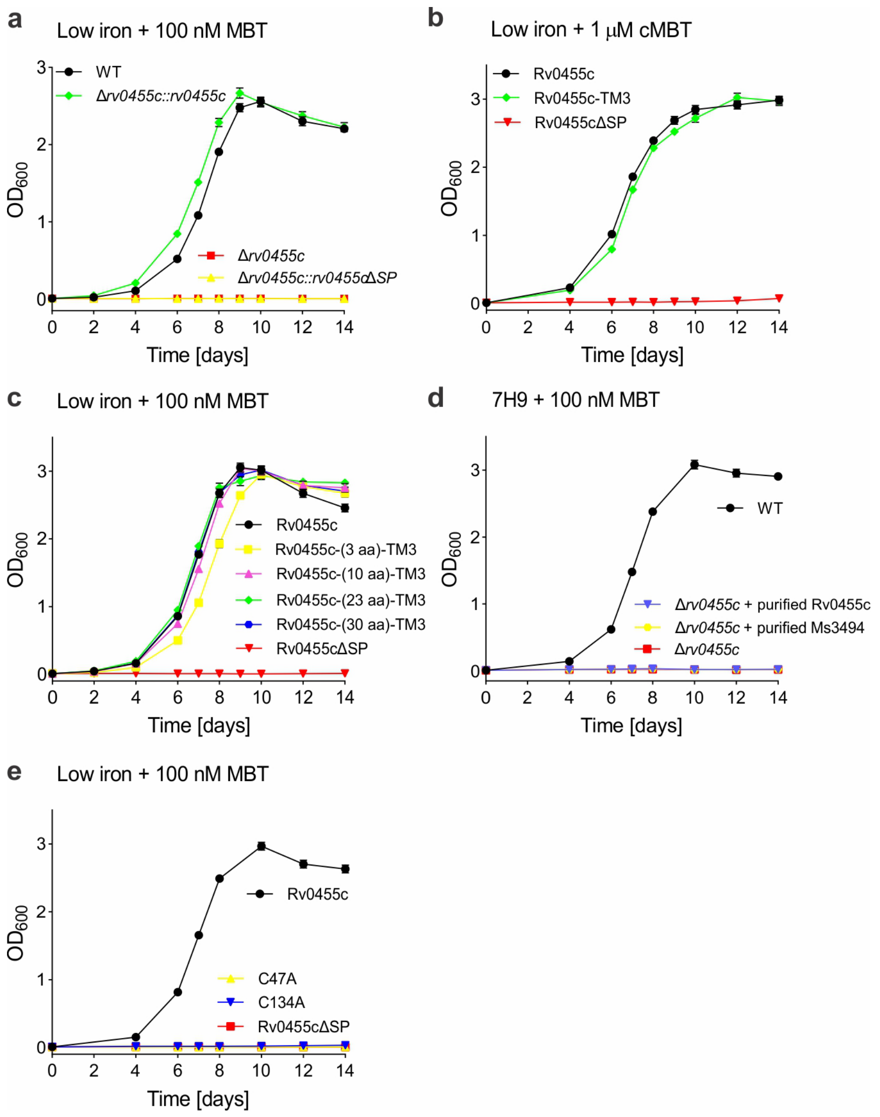

Figure S6. Growth assays of the Mtb Δ*rv0455c* mutant complemented with *rv0455c* variants.
a. Growth curves of Mtb strains including Mtb mc^{2}6230 (as wt), the Δ*rv0455c* deletion mutant (Mtb ML2203), and the Δ*rv0455c* mutant complemented with intact *rv0455c* (Mtb ML2205) or the signal sequence removed *rv0455c*$_{31\text{-}148}$ (Mtb ML2206), in 7H9 low iron (less than 0.1 μM Fe^{3+}) medium supplemented with 100 nM Fe-MBT. **b.** Growth curves of Δ*rv0455c* mutant complemented with the gene encoding membrane-anchored Rv0455c-TM3 (ML2778) in low iron 7H9 medium supplemented with 1 μM Fe-cMBT. **c.** Growth curves of Δ*rv0455c* mutants complemented with genes encoding membrane-anchored Rv0455c proteins with different length of the linkers (3-, 10-, 23-, 30-amino acid) between the C-terminus of Rv0455c and the transmembrane helices (TM3) in low iron 7H9 medium supplemented with 100 nM Fe-MBT. **d.** Growth curves of Δ*rv0455c* mutant in the presence of 1 μM purified Rv0455c$_{31\text{-}148}$ protein or 2 μM purified MSMEG_3494$_{33\text{-}153}$ protein in 7H9 iron-replete medium supplemented with 100 nM Fe-MBT. **e.** Growth curves of Δ*rv0455c* mutants complemented with genes encoding cysteines mutated Rv0455c in low iron 7H9 medium supplemented with 100 nM Fe-MBT. The Mtb ML2205 and ML2206 strains were used as positive control and negative control, respectively, in (**b**), (**c**), (**e**). The initial OD_{600} of all cultures is 0.01. Error bars represent standard deviations from the mean results of biological triplicates (*n* = 3). Source data are provided in the Source Data file.

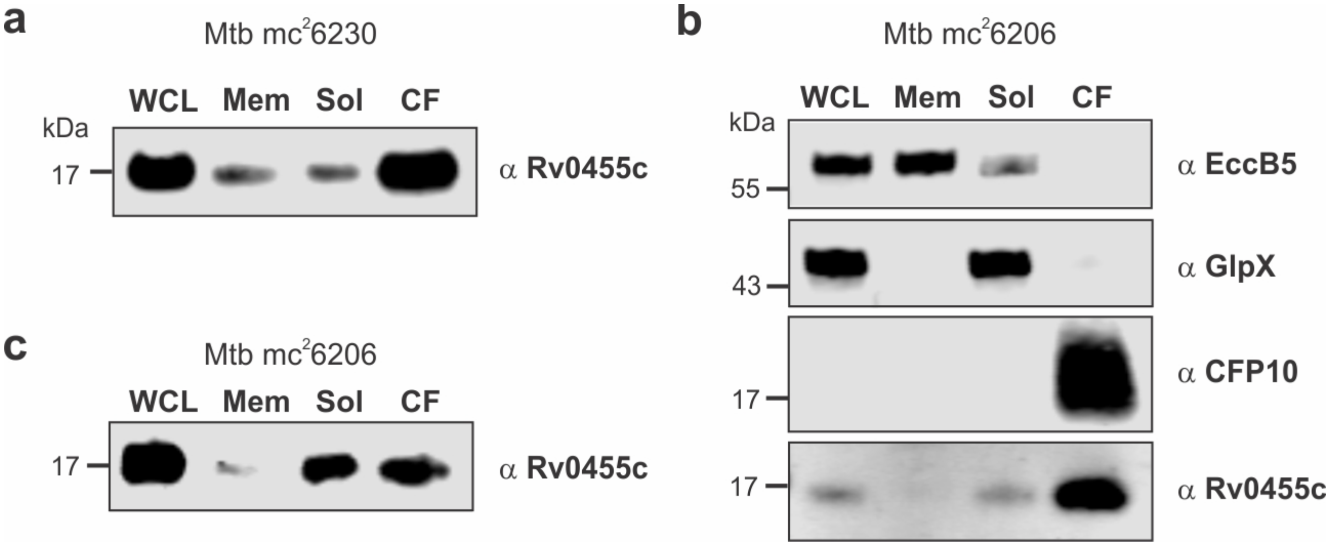

Figure S7. Subcellular localization of Rv0455c in *M. tuberculosis*.
Immunoblot analysis of whole-cell lysates (WCL), membrane-associated pellet (Mem), water-soluble supernatant (Sol), and culture filtrate (CF) from Mtb mc^{2}6230 (**a**) and from Mtb mc^{2}6206 (**b**, **c**). The culture filtrate was concentrated 200-fold. Rv0455c was detected with an Rv0455c-specific antiserum. EccB5, GlpX, and CFP10 served as indicator proteins for the membrane-associated proteins, soluble proteins and secreted proteins, respectively. 5 µL of each fraction taken from the WCL, Mem2, Sol2 and CF was analyzed by 8% SDS-PAGE gels and Western blots as shown in (**b**). The quantitative image analysis of western blots for Rv0455c was performed using (**a**) and (**c**) generated from 8% SDS-PAGE gels loaded with 20 µL of the WCL, Mem, Sol fractions and 5 µL of the CF. Data were obtained from at least two independent experiments and representative images are shown. Source data are provided in the Source Data file.

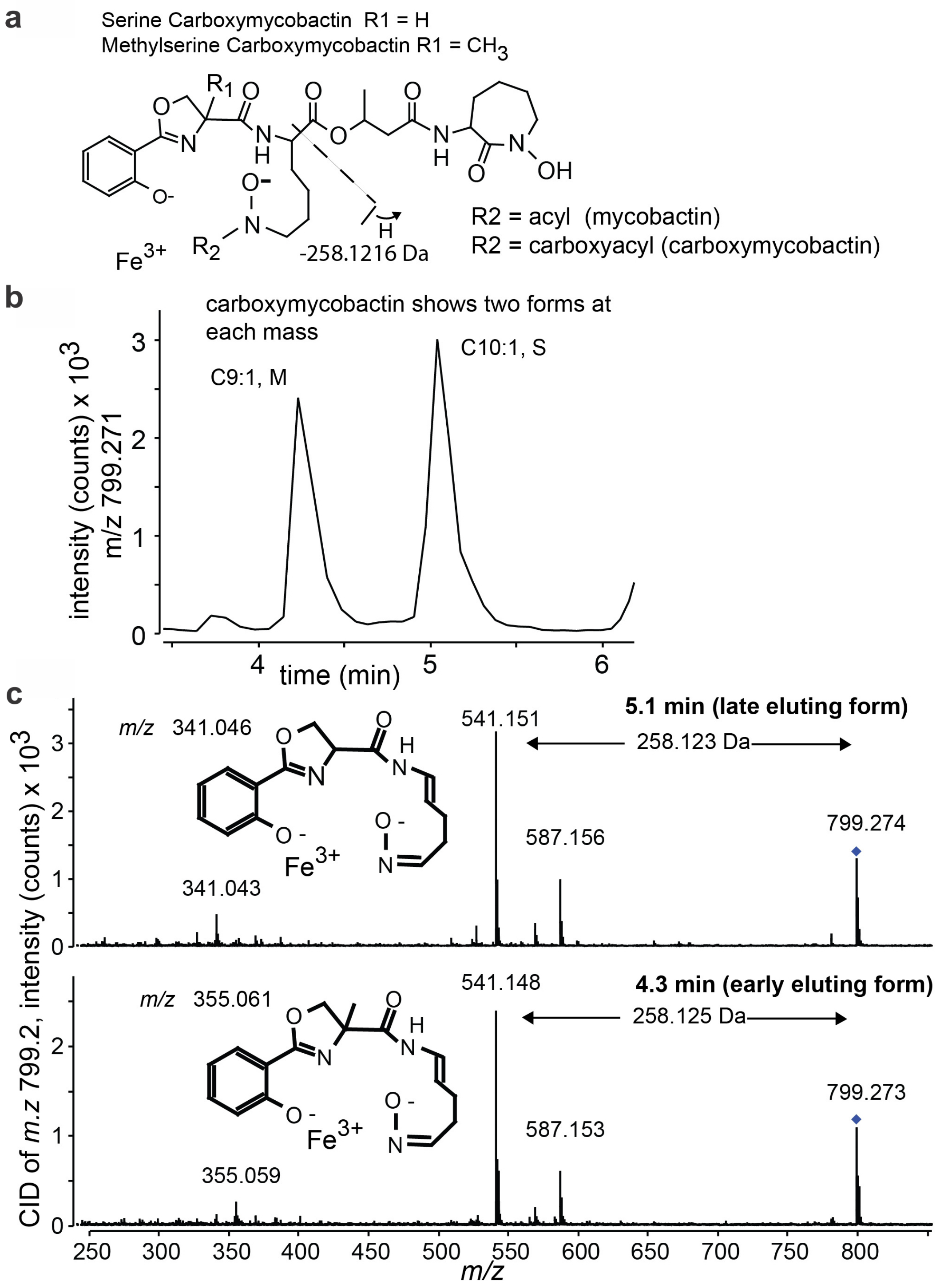

Figure S8. Siderophores analyzed by high-resolution mass spectrometry. Carboxymycobactin and mycobactin species vary at two positions (**a**) with either serine or methylserine at the R_1 position and different chain lengths and double bond number at the R_2 position. Thus they yielded two major peaks for every calculated m/z value (**b**). Collision-induced dissociation (CID) of the molecular ion (**c**) yields major ions showing loss of the cobactin group (right side) and giving exactly the same mass for two forms. A lower intensity ion at either m/z 341 or m/z 355 is consistent with a secondary fragmentation to lose the R_2 group (and a hydrogen) allowing assignment of the early peak as the methylserine form and the later eluting peak as the serine form.

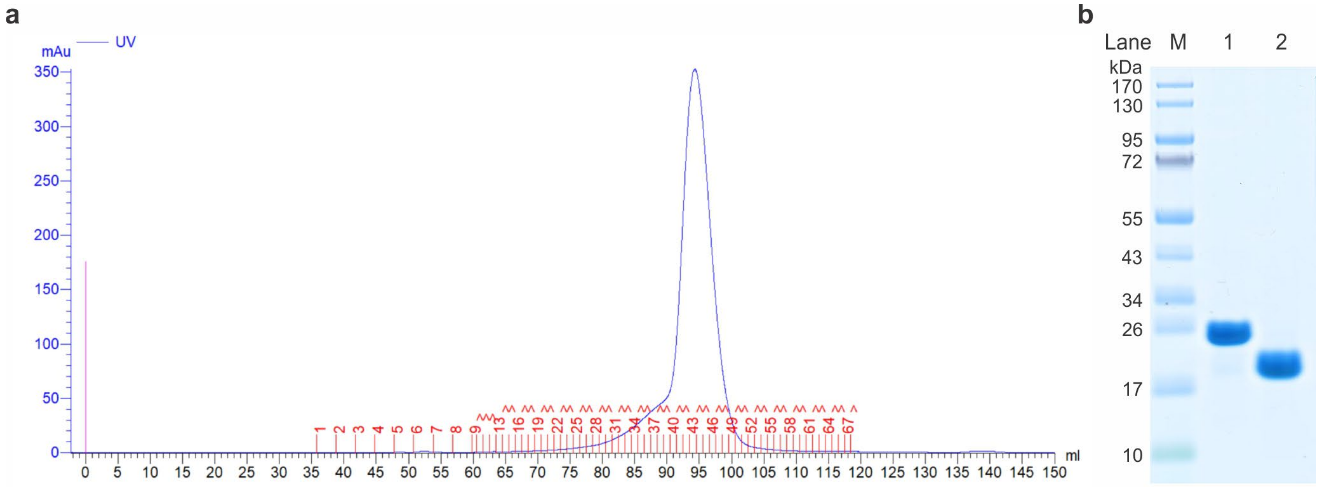

Figure S9. Purification and analysis of recombinant MSMEG_3494 protein.

a. Size exclusion chromatography of the MSMEG_3494$_{33\text{-}153}$ protein after removal of the affinity tag by enterokinase. The column material was Superdex 75. The fractions from 39 to 49 were pooled and analyzed by gel electrophoresis. **b.** Analysis of purified recombinant MSMEG_3494$_{33\text{-}153}$ protein. Lanes: M, protein marker; 1, MSMEG_3494$_{33\text{-}153}$ protein with a C-terminal StrepII-His$_8$ tag after Ni(II) affinity chromatography; 2, MSMEG_3494$_{33\text{-}153}$ protein after removal of the StrepII-His$_8$ tag by enterokinase and subsequent purification by size exclusion chromatography as shown in Fig. S9a.

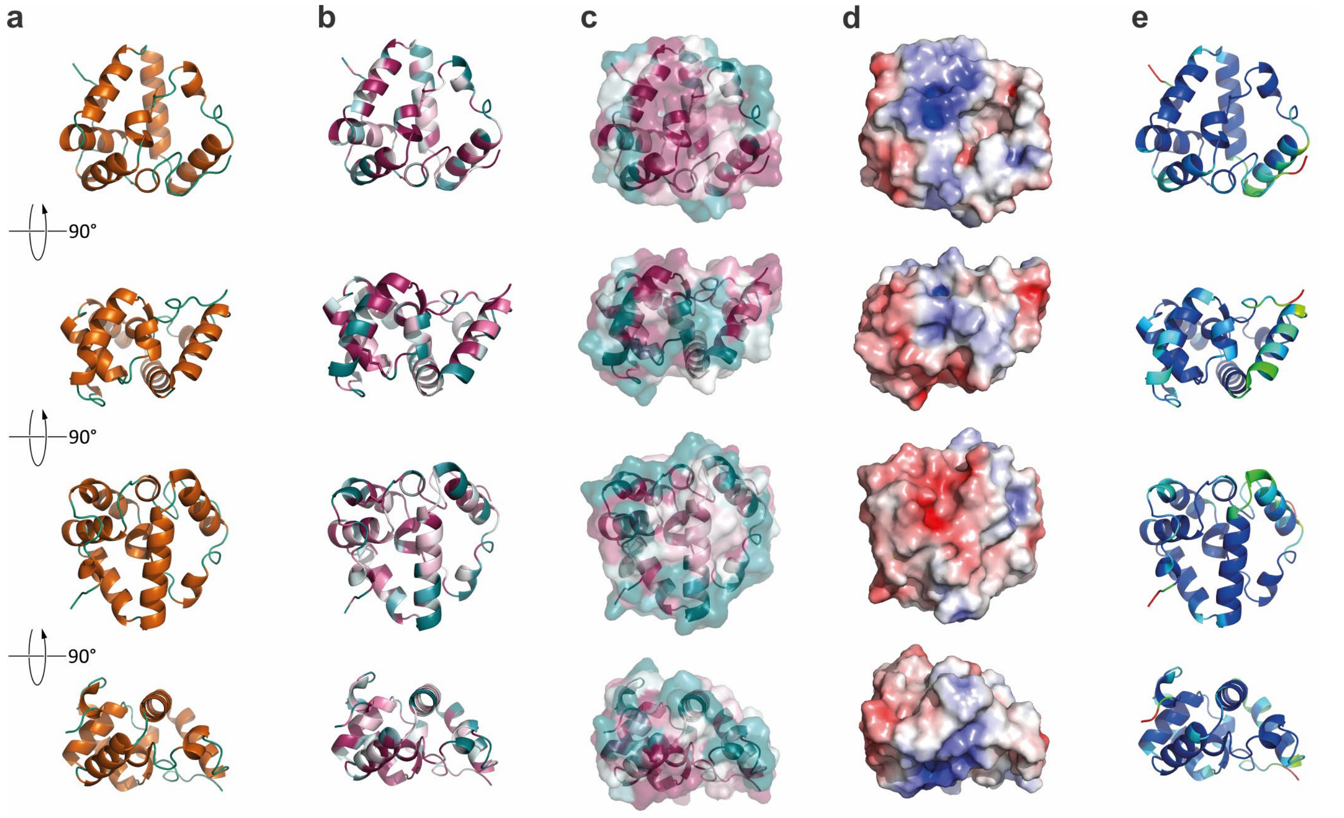

Figure S10. Orthogonal views of MSMEG_3494

a. Colors reflect the secondary structure, depicting alpha helixes in orange and loops in green. **b.** cartoon depiction. **c.** transparent surface depiction with cartoon shown. Colors reflect the conservation of amino acids across the 150 closest members of DUF5078 to MSMEG_3494. Purple indicates high conservation, white indicates moderate conservation, and teal indicates a lack of conservation. **d.** Surface depiction of the electrostatic surface potential from -5 kT/e (red) to +5 kT/e (blue). **e.** Colors reflect the *B*-factor calculated for each residue. Spectrum spanning 20Å^2 (blue) to 50Å^2 (red).

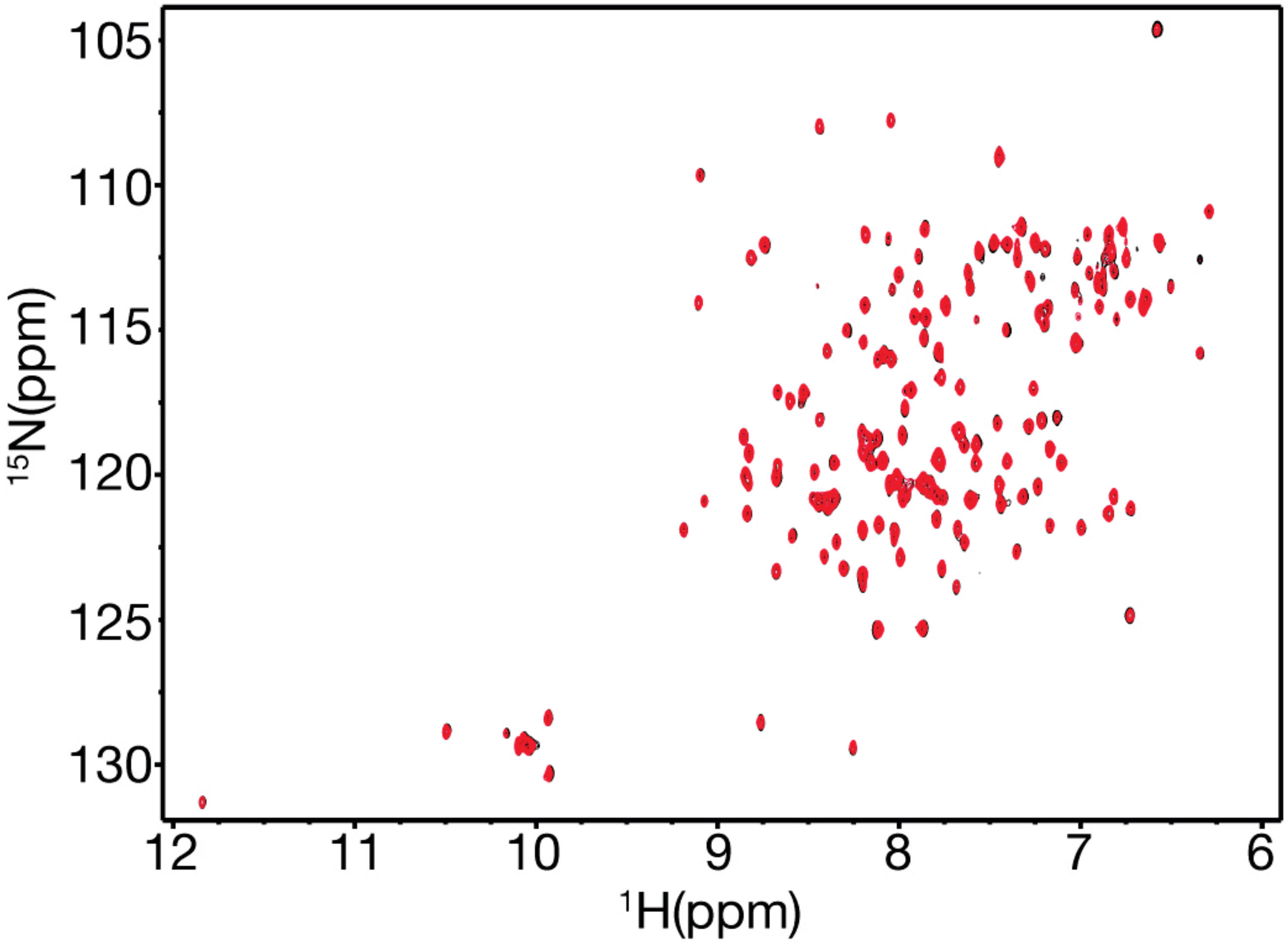

Figure S11. NMR spectra of purified MSMEG_3494 with and without carboxymycobactin. Overlaid spectra of ^{15}N-labeled MSMEG_3494$_{33\text{-}153}$ protein (50 µM) with (red) and without (black) 100 µM deferrated carboxymycobactin from a 2D ^{15}N,^{1}H-HSQC experiment. All experiments were acquired in 50 mM sodium phosphate (pH 6.5) at 25 °C on a Bruker Avance II (700 MHz) NMR spectrometer. Data were obtained from at least two independent experiments and representative images are shown.

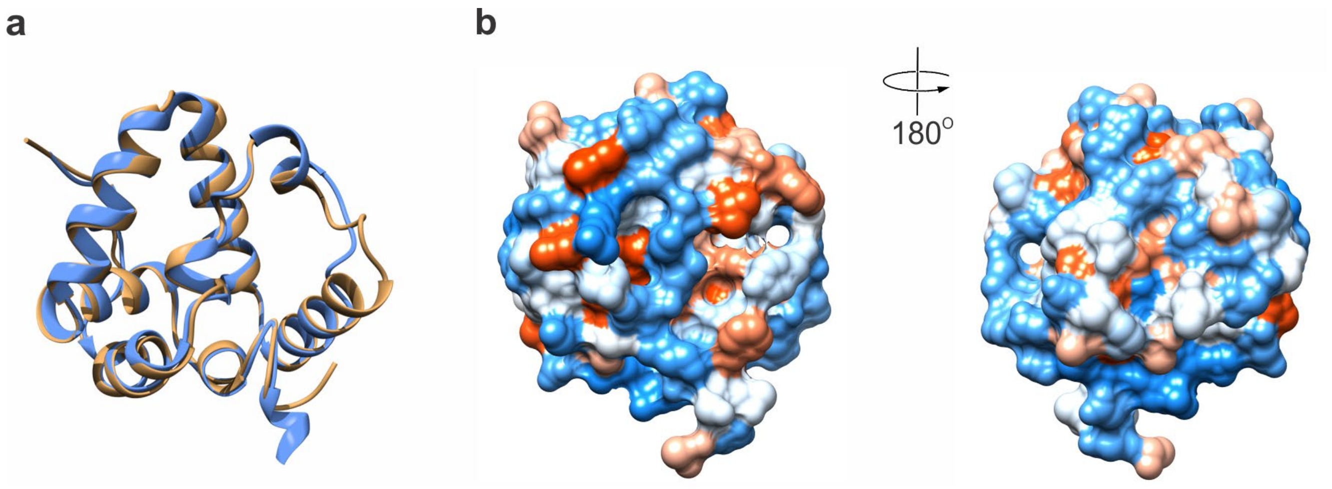

Figure S12. Predicted structure of Rv0455c from *M. tuberculosis*.

a. Overlay of the crystal structure of MSMEG_3494 with the predicted structure of Rv0455c. The crystal structure of *M. smegmatis* MSMEG_3494 (Khaki, PDB ID: 7REF) was aligned with a model of *M. tuberculosis* Rv0455c obtained from the AlphaFold Database (blue, ID: AF-O53740[1] using UCSF Chimera[2]. The root-mean-square deviation (RMSD) of both structures is 0.5 Å. The N-terminal signal peptide (residues 1-30) was truncated in both structures. **b.** View of the hydrophobicity surface of the predicted Rv0455c structure. Hydrophobic and charged residues are colored with orange and blue, respectively. The N-terminal signal peptide (residues 1-30) was truncated.

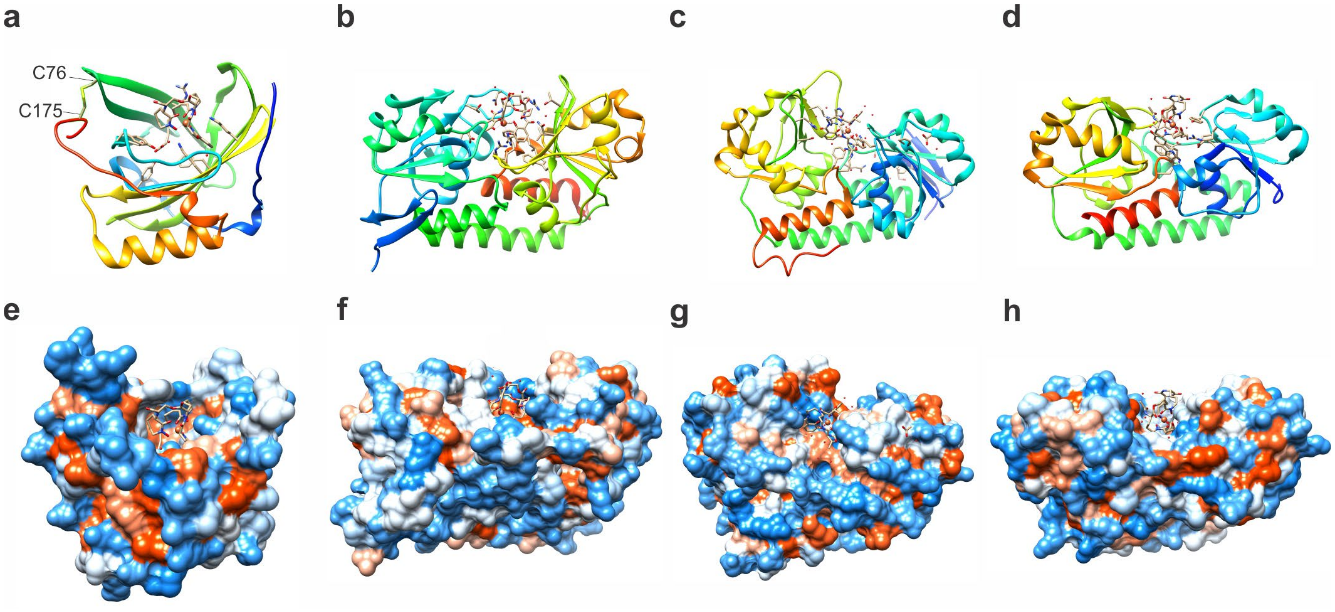

Figure S13. Structures of protein-siderophore complexes.
Structures of siderocalin (PDB ID: 1X8U)[3] of human, FepB (PDB ID: 3TLK) of *E. coli*[4], ViuP (PDB ID: 3R5T) of *Vibrio cholera*[5], and FhuD (PDB ID: 1ESZ) of *E. coli*[6], complexed with ferric carboxymycobactin T, ferric enterobactin, ferric vibriobactin and ferric coprogen, respectively. **a-d.** Ribbon representation of the siderophore-binding proteins siderocalin (**a**), FepB (**b**), ViuP (**c**) and FhuD (**d**) colored by amino acid sequence from the N-terminus (blue) to the C-terminus (red). The two cysteines of siderocalin are indicated. **e-h.** The view of the hydrophobicity surface of siderocalin (**e**), FepB (**f**), ViuP (**g**) and FhuD (**h**). The hydrophobic residues and the charged residues are indicated with orange and blue, respectively. The structures were visualized with UCSF Chimera 1.14[2].

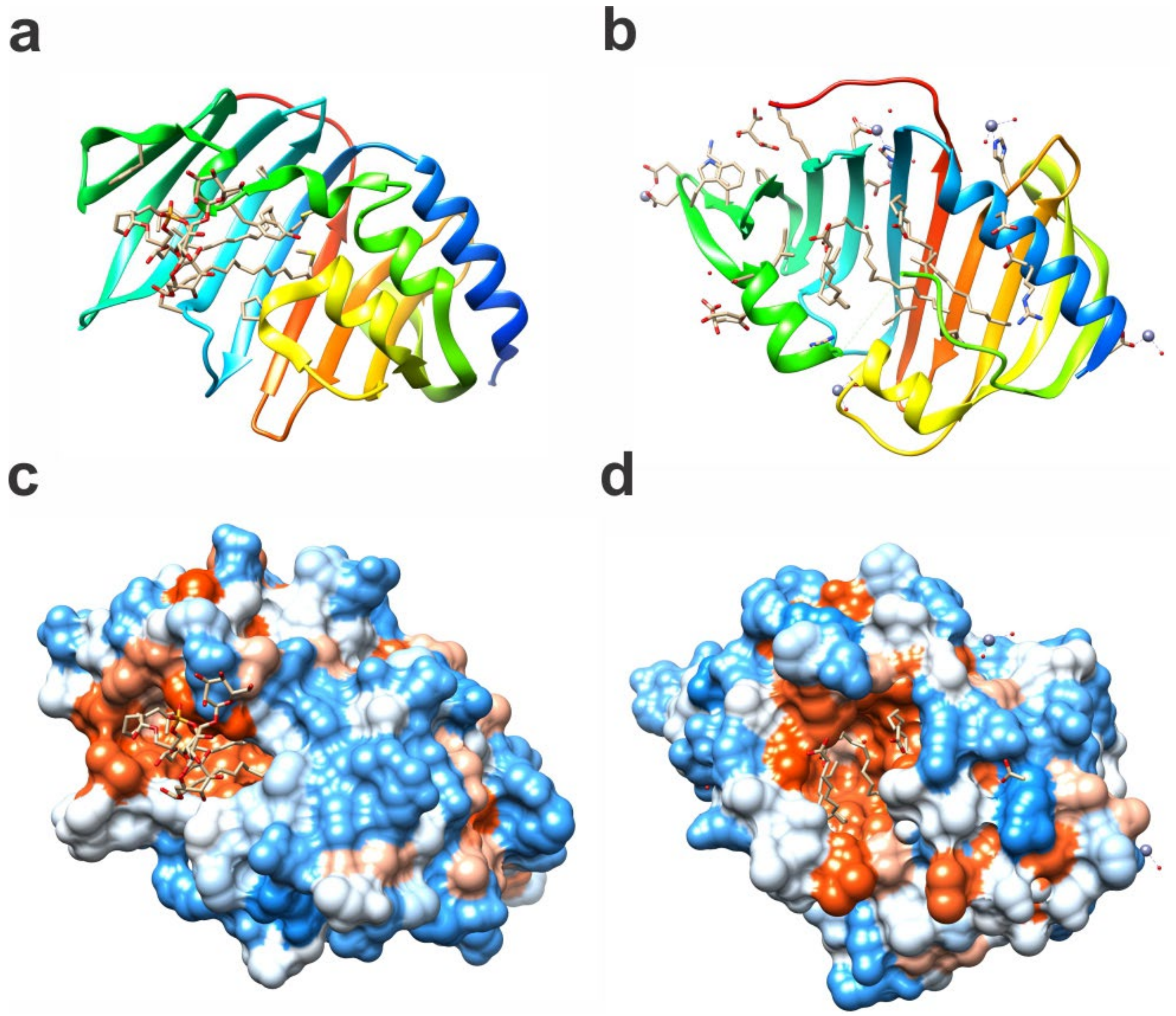

Figure S14. Structures of the Mtb lipoproteins LprG and LppX.

Structures of the Mtb lipoproteins LprG (PDB ID: 3MHA)[7] and LppX (PDB ID: 2BYO)[8] with lipid substrates. **a, b.** Ribbon representation of the lipoproteins LprG (**a**) and LppX (**b**) colored by amino acid sequence from the N-terminus (blue) to the C-terminus (red). **c, d.** The view of the hydrophobicity surface of LprG (**c**) and LppX (**d**). The hydrophobic residues and the charged residues are indicated with orange and blue, respectively. The structures were visualized with UCSF Chimera 1.14[2].

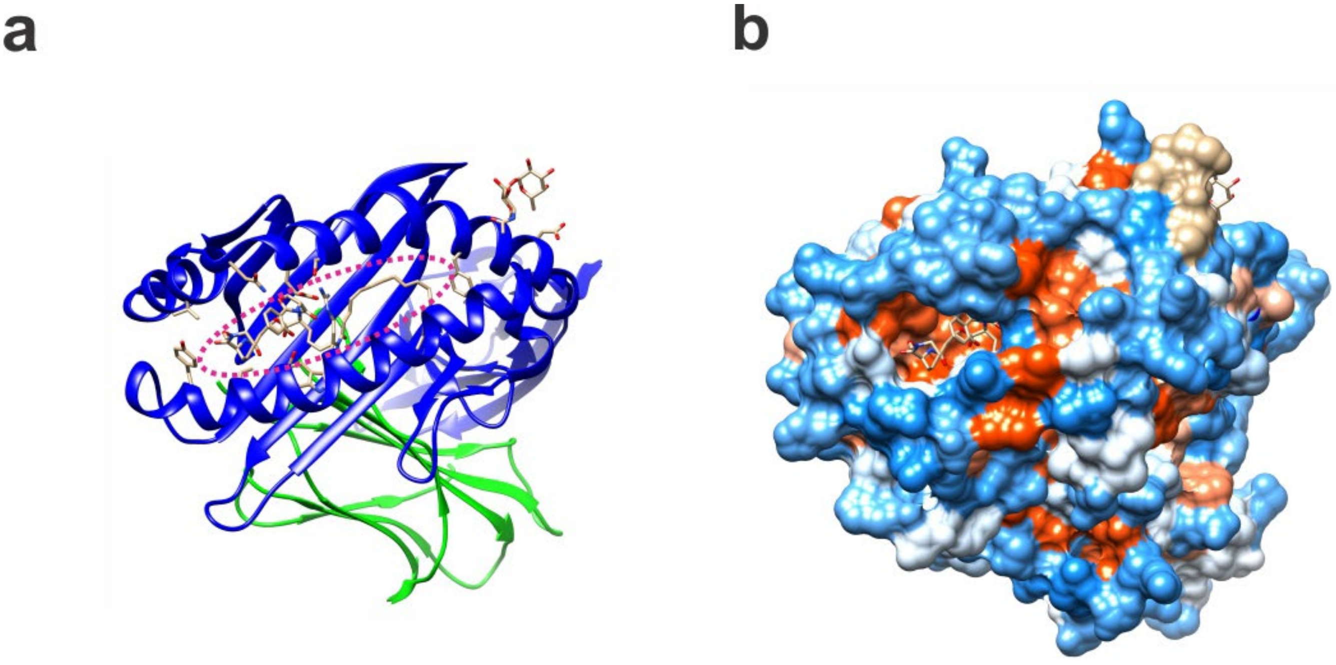

Figure S15. Structure of the human CD1a complexed with a mycobactin analog.
Structure of the human CD1a complexed with a synthetic mycobactin lipopeptide (PDB ID: 1XZ0)[9]. **a.** Cartoon representation of the CD1a (α1–α3 domains) (blue) - β_2M (green) heterodimer with bound mycobactin analog circled in pink. **b.** View of the hydrophobicity surface of the CD1a complex. The hydrophobic residues and the charged residues are indicated with orange and blue, respectively. The structures were visualized with UCSF Chimera 1.14[2].

SUPPLEMENTARY TABLES

Strain	Relevant genotypes and description	Source or reference
E. coli DH5α	F⁻ Φ80*lacZ*Δ*M15* Δ*(lacZYA-argF)* U169 *recA1 endA1 hsd*R17*(rk-, mk+) phoA sup*E44 *thi-1 gyrA96 relA1* λ⁻	Invitrogen
E. coli BL21 (DE3)	*fhuA2 [lon] ompT gal (λ DE3) [dcm]* Δ*hsdS λ DE3 = λ sBamHIo* Δ*EcoRI-B int::(lacI::PlacUV5::T7 gene1) i21* Δ*nin5*	NEB
M. tuberculosis mc²6230	H37Rv derivative; ΔRD1 Δ*panCD*; avirulent	[10]
M. tuberculosis mc²6206	H37Rv derivative; Δ*leuCD* Δ*panCD*; avirulent	[11]
M. tuberculosis ML859	mc²6230 derivative; Δ*mmpS4*::*loxP,* Δ*mmpS5::loxP*	[12]
M. tuberculosis ML1600	mc²6230 derivative; Δ*mbtD*::*hyg*^{R} (*rv2381c* deletion)	[13]
M. tuberculosis ML2203	mc²6230 derivative; Δ*rv0455c*::*hyg*^{R}	This study
M. tuberculosis ML2205	mc²6230 derivative; Δ*rv0455*::*hyg*^{R}, L5 attB::pML3613 (P_{smyc}::*rv0455c*), *kan*^{R}	This study
M. tuberculosis ML2206	mc²6230 derivative; Δ*rv0455*::*hyg*^{R}, L5 attB::pML3614 (P_{smyc}::*rv0455c*$_{31-148}$), *kan*^{R}	This study
M. tuberculosis ML2290	mc²6230 derivative; Δ*rv0455*::*hyg*^{R}, L5 attB::pML4274 (P_{smyc}::*rv0455c_C47A*), *kan*^{R}	This study
M. tuberculosis ML2291	mc²6230 derivative; Δ*rv0455*::*hyg*^{R}, L5 attB::pML4275 (P_{smyc}::*rv0455c_C134A*), *kan*^{R}	This study
M. tuberculosis ML2293	mc²6230 derivative; Δ*rv0455*::*hyg*^{R}, L5 attB::pML4279 (P_{smyc}::*MSMEG_3494-strepII-his8*), *kan*^{R}	This study
M. tuberculosis ML2294	mc²6230 derivative; Δ*rv0455*::*hyg*^{R}, L5 attB::pML4280 (P_{smyc}::*ML2380*), *kan*^{R}	This study
M. tuberculosis ML2295	mc²6230 derivative; Δ*rv0455*::*hyg*^{R}, L5 attB::pML4281 (P_{smyc}::*B586_19750*), *kan*^{R}	This study
M. tuberculosis ML2298	mc²6230 derivative; Δ*rv0455*::*hyg*^{R}, L5 attB::pML4284 (P_{smyc}::*rv0455c_Twin-strep*), *kan*^{R}	This study
M. tuberculosis ML2299	mc²6230 derivative; Δ*rv0455*::*hyg*^{R}, L5 attB::pML4285 (P_{smyc}::*rv0455c_GS linker_MBP*), *kan*^{R}	This study
M. tuberculosis ML2770	mc²6230 derivative; Δ*rv0455*::*hyg*^{R}, L5 attB::pML4605 (P_{smyc}::*MSMEG_3494*), *kan*^{R}	This study
M. tuberculosis ML2777	mc²6230 derivative; Δ*rv0455*::*hyg*^{R}, L5 attB::pML4601 (P_{smyc}::*rv0455c_GS linker (3-aa)_TM3*), *kan*^{R}	This study
M. tuberculosis ML2778	mc²6230 derivative; Δ*rv0455*::*hyg*^{R}, L5 attB::pML4299 (P_{smyc}::*rv0455c_GS linker (10-aa)_TM3*), *kan*^{R}	This study
M. tuberculosis ML2779	mc²6230 derivative; Δ*rv0455*::*hyg*^{R}, L5 attB::pML4602 (P_{smyc}::*rv0455c_GS linker (23-aa)_TM3*), *kan*^{R}	This study
M. tuberculosis ML2780	mc²6230 derivative; Δ*rv0455*::*hyg*^{R}, L5 attB::pML4603 (P_{smyc}::*rv0455c_GS linker (30-aa)_TM3*), *kan*^{R}	This study
M. smegmatis mc²155	wild-type, laboratory strain	[14]

M. smegmatis ML2248	mc²155 derivative; Δ*fxbA*::*hyg*^{R} (*msmeg_0014* deletion)	This study
M. smegmatis ML2249	mc²155 derivative; Δ*fxbA*::*loxP*	This study
M. smegmatis ML2275	mc²155 derivative; Δ*fxbA*::*loxP,* Δ*msmeg_3494::hyg*^{R}	This study
M. smegmatis ML2276	mc²155 derivative; Δ*fxbA*::*loxP,* Δ*msmeg_3494::hyg*^{R}, L5 attB::pML3613 (P_{smyc}*::rv0455c*), *kan*^{R}	This study
M. tuberculosis H37Rv	H37Rv, wild-type, laboratory strain	ATCC 25618
M. tuberculosis ML2710	H37Rv, L5 attB::pCV125, *kan*^{R}	This study
M. tuberculosis ML2700	H37Rv derivative; Δ*rv0455c*::*hyg*^{R}	This study
M. tuberculosis ML2701	H37Rv derivative; Δ*rv0455c*::*hyg*^{R}, L5 attB::pML3613 (P_{smyc}*::rv0455c*), *kan*^{R}	This study
M. tuberculosis ML2711	H37Rv derivative; Δ*rv0455c*::*hyg*^{R}, L5 attB::pCV125, *kan*^{R}	This study

Table S1. Bacterial strains used in this work.
The annotations hyg^R and kan^R indicate that the strain is resistant to the antibiotics hygromycin and kanamycin, respectively.

Plasmid	Components and properties	Source or reference
pML2714	pUC origin; pAL5000ts; P_{hsp60}::*cre*; P_{imyc}::*pamcherry1m*; *aph*; 7613 bp	[15]
pML3694	pUC origin; pAL5000ts; *sacR*; *sacB*; *tdTomato*; up_*rv0455c*_hom; *loxP*-P_{smyc}::*gfp-hyg-loxP*; down_*rv0455c*_hom; 11525 bp	This study
pML3655	pUC origin; *sacR*; *sacB*; *tdTomato*; up_*fxbA*_hom; *loxP*-P_{smyc}::*gfp-hyg-loxP*; down_*fxbA*_hom; 9166 bp	This study
pML3618	pUC origin; sacR; sacB; *tdTomato*; up_*MSMEG_3494*_hom; *loxP*-P_{smyc}::*gfp-hyg-loxP*; down_*MSMEG_3494*_hom; 9360 bp	This study
pCV125	ColE1 origin; *int;* L5 *attP; aph; lacZ;* 8261 bp	[16]
pML3613	pCV125 derivative; ColE1 origin; *int*; L5 *attP*; *aph*; p_{smyc}::*rv0455c*; 5827 bp	This study
pML3614	pCV125 derivative; ColE1 origin; *int*; L5 *attP*; *aph*; p_{smyc}::$rv0455c_{31-148}$ (signal sequence deleted); 5740 bp	This study
pML4274	pCV125 derivative; ColE1 origin; *int*; L5 *attP*; *aph*; p_{smyc}::*rv0455c_C47A* (Cys47 to Ala mutation); 5827 bp	This study
pML4275	pCV125 derivative; ColE1 origin; *int*; L5 *attP*; *aph*; p_{smyc}::*rv0455c_C134A* (Cys134 to Ala mutation); 5827 bp	This study
pML4279	pCV125 derivative; ColE1 origin; *int*; L5 *attP*; *aph*; p_{smyc}::*MSMEG_3494-*D_4K*-strepII-*his_8; 5905 bp	This study
pML4280	pCV125 derivative; ColE1 origin; *int*; L5 *attP*; *aph*; p_{smyc}::*ML2380*; 5842 bp	This study
pML4281	pCV125 derivative; ColE1 origin; *int*; L5 *attP*; *aph*; p_{smyc}::*B586_19750*; 5839 bp	This study
pML4284	pCV125 derivative; ColE1 origin; *int*; L5 *attP*; *aph*; p_{smyc}::*rv0455c_twin-strep*; 5921 bp	This study
pML4285	pCV125 derivative; ColE1 origin; *int*; L5 *attP*; *aph*; p_{smyc}::*rv0455c_GS linker (20-aa)-MBP*; 6995 bp	This study
pML4601	pCV125 derivative; ColE1 origin; *int*; L5 *attP*; *aph*; p_{smyc}::*rv0455c_GS linker (3-aa)-3 TM helices*; 6098 bp	This study
pML4299	pCV125 derivative; ColE1 origin; *int*; L5 *attP*; *aph*; p_{smyc}::*rv0455c_GS linker (10-aa)-3 TM helices*; 6119 bp	This study
pML4602	pCV125 derivative; ColE1 origin; *int*; L5 *attP*; *aph*; p_{smyc}::*rv0455c_GS linker (23-aa)-3 TM helices*; 6158 bp	This study
pML4603	pCV125 derivative; ColE1 origin; *int*; L5 *attP*; *aph*; p_{smyc}::*rv0455c_GS linker (30-aa)-3 TM helices; 6179 bp*	This study
pET22b	pBR322 origin; *lacI*; p_{T7} promoter; *bla*; 5493 bp	Novagen
pML4212	pET22b derivative; pBR322 origin; *lacI*; p_{T7}::$SS_{OmpF(1\text{-}23\ aa)}$-$rv0455c_{31-148}$-$his_6$; *bla*; 5824 bp	This study
pML4249	pET22b derivative; pBR322 origin; *lacI*; p_{T7}::$SS_{OmpF(1\text{-}23\ aa)}$-$MSMEG\_3494_{33-153}$-$D_4K$-*strepII*-$his_8$; *bla*; 5878 bp	This study
pML4604	pET22b derivative; pBR322 origin; *lacI*; p_{T7}::$SS_{dsbC(1\text{-}20\ aa)}$-$MSMEG\_3494_{33-153}$-$D_4K$-*strepII*-$his_8$; *bla*; 5869 bp	This study

Table S2. plasmids used in this work.

Up- and downstream homologous sequences of genes are subscripted as up and down. “Origin” denotes origin of replication. The genes *hyg, aph* and *bla* confer resistance to hygromycin, kanamycin and Ampicillin, respectively.

Oligonucleotide	Sequence (5' to 3')	Use
Rv0455c_UP-f	AGAGATACTAGTTGAGACACTTGACCCTTAGG	Gene deletion, pCR validation
Rv0455c_UP-r	CTCTCAATTTAAATCATAGTGGTTACCGTAGCAC	Gene deletion
Rv0455c_DN-f	CACACTTAATTAAGGTGTGGAACTGGGCCTAA	Gene deletion
Rv0455c_DN-r	ATATATATGCATGTCCAGGCATCGACTTCGAC	Gene deletion, pCR validation
fxbA_UP-f	AGATATAACTAGTCAGCGGTAGATGGATGCTC	Gene deletion, pCR validation
fxbA_UP-r	GGAGAGATTTAAATCCAGCTTTCTGGCAGGTT	Gene deletion
fxbA_DN-f	AGAGAGTTAATTAAATACCTGACCGCCACACC	Gene deletion
fxbA_DN-r	AGATATATATGCATCCCAGCATCTGTTCACGG	Gene deletion, pCR validation
Ms3494_UP-f	AGAGATACTAGTGTCCATGCGATCGGAGACCT	Gene deletion, pCR validation
Ms3494_UP-r	CTCTCAATTTAAATGATATCGCTCCAAAGATGTC	Gene deletion
Ms3494_DN-f	CACACTTAATTAATGTCCGTCTGGAACTGGTAG	Gene deletion
Ms3494_DN-r	ATATATATGCATTCGTCGGGACTCATCGACAC	Gene deletion, pCR validation
Rv0455c_inner-f	GACTCCACGGAAGACTTTCC	PCR validation
fxbA_inner-f	GGAATCGAGGTGCTCCTGGCCAAGC	PCR validation
Ms3494_inner-f	CCGAACCGGATCATGAGAACG	PCR validation
gfp-r	CGTCTTGTAGTTACCGTCGTCCT	PCR validation
hyg-f	GTCGACTTCACCGACGTCTATG	PCR validation
0455c_PacI_f	AGAGTTAATTAACAGAAAGGAGGTTAATAATGTCTCGGCTGAGTTCCAT	Expression
0455cΔSP_PacI-f	AGAGTTAATTAACAGAAAGGAGGTTAATAATGGACTCCACGGAAGACTTTCC	Expression
0455_Kpn-r	ATATGGTACCTTAGGCCCAGTTCCACACCGACA	Expression
Ms3494_Pac-f	AGAGTTAATTAACAGAAAGGAGGTTAATAGTGAAACGCATGCGTGTGAA	Expression
3494_Kpn-r	ATATGGTACCCTACCAGTTCCAGACGGACA	Expression
ML2380_PacI-f (B586_19750-f)	AGAGTTAATTAACAGAAAGGAGGTTAATAATGTCTCGGCTGAGCACC	Expression
ML2380_KpnI-r (B586_19750-r)	ATATGGTACCCTACGGCCAGTTCCACAC	Expression
Rv0455_C47A-r	AGATATTGTTCGGCGTC**AGC**GGTGGTTGCGATCATCC	Point mutation
Rv0455_C47A-f	GGATGATCGCAACCACC**GCT**GACGCCGAACAATATCT	Point mutation
Rv0455_C134A-r	CCAGTTCCACACCGACATGTCGCCGGCTGGGTATCCATT**AGC**CACCTCGGTCCCTTTAGC	Point mutation
Ms3494_33-f	TCCCTGCTCTGTTAGTAGCAGGTACTGCAAACGCTGCAGATGCCACCGACGACTAC	Expression
0455_TwinStrep-r	ATATGCGGCCGCAAGCTTACTTCTCGAACTGCGGGTGGGACCAGGCCGAGCTGCCACCCGAGCCACCGCCGCTGCCACCGCCCTTCTCGAACTGCGGGTGCGACCAGGTACCGGCCCAGTTCCACACCGACA	Expression

GS linker-f	ATATGGTACCGGCGGTGGCAGCGGTGGCGGTTCGGGTGGCGGTTCCGGTGGCAGCTCGCA	Expression
MBP_Nde-f	GGTTCCGGTGGCAGCTCGCATATGAAAATCGAAGAAGGTAAACTGG	Expression
MBP_Not-r	ATATGCGGCCGCAAGCTTACTTGGTGATACGAGTCTG	Expression
TM3_Kpn-f	ATATAGGTACCGGTGGCGGTTCGGGTGGCGGTAGTCTGCAGGTGATCGAG	Expression
TM3_Nde-Kpn-f	ATATATATCATATGGGTACCAGTCTGCAGGTGATCGAG	Expression
TM3_Nde-30-f	ATATATATCATATGGGTGGCGGTTCGGGTGGCAGTGGTGGTAGTCTGCAGGTGATCGAG	Expression
TM3_Hind-r	ATATATAAGCTTAGAGCCCGCGTGCTTCCTGGTAG	Expression
SSompF_Nde-f	ATATATATCATATGATGAAGCGCAATATTCTGGCAGTGATCGTCCCTGCTCTGTTAGTAG	Expression in E. coli
0455_31-f	TCCCTGCTCTGTTAGTAGCAGGTACTGCAAACGCTGCAGACTCCACGGAAGACTTTCC	Expression in E. coli
0455His6_Hind-r	ATATAAGCTTAGTGGTGGTGGTGGTGGTGGGCCCAGTTCCACACCGACAT	Expression in E. coli
SSompF-3494_33-f	TCCCTGCTCTGTTAGTAGCAGGTACTGCAAACGCTGCAGATGCCACCGACGACTAC	Expression in E. coli
SSdsbC_Nde-f	ATATATATCATATGAAGAAAGGTTTTATGTTGTTTACTTTGTTAGCGGCGTTTTCAGGCT	Expression in E. coli
dsbC-3494_33-f	TAGCGGCGTTTTCAGGCTTTGCTCAGGCTGATGCCACCGACGACTAC	Expression in E. coli
3494-D4K-r	GGTGAGACCATTTGTCATCGTCATCCCAGTTCCAGACGGACA	Expression in E. coli
StrepHis8_Hind-r	ATATAAGCTTAGTGGTGGTGGTGGTGGTGGTGGTGCTTCTCGAACTGCGGGTGAGACCA	Expression in E. coli
TM3 region	CTGCAGGTGATCGAGGCGGTCACGTTCACCGTGATCATCGTGATGTTGCTGCTGGTCTACCGGTCGATCATCACGTCGGCGATCATGCTGACGATGGTGGTGCTCGGGCTGCTCGCCACCCGCGGGGGCGTGGCTTTCCTCGGTTTCCACCGGATCATTGGGCTCTCGACCTTCGCGACCAACCTGCTCGTGGTGCTGGCGATCGCGGCCGCCACCGACTACGCCATCTTCCTGATCGGCCGCTACCAGGAAGCACGCGGGCTCTAA<br><br>Amino acid sequence:<br>**LQVIEAVTFTVIIVMLLLVY**RSIITS**AIMLTMVVLGLLATRGGVAFLGF**HRIIGLST**FATNLLVVLAIAAATDYAIFLIG**RYQEARGL<br>(The predicted transmembrane helices are highlighted in bold.)	Expression
GS linkers	3-aa linker: GGTACCAGT (amino acid sequence: GTS)<br><br>10-aa linker: GGTACCGGTGGCGGTTCGGGTGGCGGTAGT (amino acid sequence: GTGGGSGGGS)<br><br>23-aa liner:<br>GGTACCGGCGGTGGCAGCGGTGGCGGTTCGGGTGGCGGTTCCGGTGGCAGCTCGCATATGGGTACCAGT<br>(amino acid sequence: GTGGGSGGGSGGGSGGSSHMGTS)<br><br>30-aa linker:<br>GGTACCGGCGGTGGCAGCGGTGGCGGTTCGGGTGGCGGTTCCGGTGGCAGCTCGCATATGGGTGGCGGTTCGGGTGGCAGTGGTGGTAGT<br>(amino acid sequence: GTGGGSGGGSGGGSGGSSHMGGGSGGSGGS)	Expression

Table S3. Oligonucleotides used in this work.
Restriction sites are underlined.

	**Peak[&]**	**Inflection**	**Remote**
**Data collection**			
Wavelength (Å)	0.91965	0.91995	0.88874
Space group	P 6		
Cell dimensions			
a, *b*, *c* (Å)	98.8285, 98.8285, 45.477		
a, b, g (°)	90, 90, 120		
Resolution (Å) *	33.46-2.10 (2.16-2.10)	33.51-2.10 (2.16-2.10)	33.51-2.10 (2.16-2.10)
R_{merge}	0.105 (0.528)	0.094 (0.527)	0.113 (0.674)
I/sI	19.4 (4.5)	17.9 (3.9)	15.1 (2.9)
Completeness (%)	99.6 (95.5)	99.8 (97.5)	99.8 (97.8)
Redundancy	10.2 (9.9)	8.1 (8.0)	8.1 (7.6)
**Refinement**			
Resolution (Å)	33.48-2.10		
R_{work}/R_{free}	0.158/0.201		
No. atoms			
Protein	2095		
Ligand/ ion	11		
Water	192		
B-factors			
Protein	25.05		
Ligand/ ion	47.92		
Water	32.41		
R.M.S. deviations			
Bond lengths (Å)	0.014		
Bond angles (°)	1.68		
Ramachandran favored/ outliers (%)	100.0/0.0		

Table S4. Data collection and refinement statistics (Multiwave Anomalous Diffraction).

* Values in parentheses are for the highest resolution shell.

[&] This data set was used for structure refinement.

SUPPLEMENTARY REFERENCES

1. Jumper, J. et al. Highly accurate protein structure prediction with AlphaFold. *Nature* **596**, 583-589 (2021).
2. Pettersen, E.F. et al. UCSF Chimera--a visualization system for exploratory research and analysis. *J Comput Chem* **25**, 1605-12 (2004).
3. Holmes, M.A., Paulsene, W., Jide, X., Ratledge, C. & Strong, R.K. Siderocalin (Lcn 2) also binds carboxymycobactins, potentially defending against mycobacterial infections through iron sequestration. *Structure* **13**, 29-41 (2005).
4. Li, B. et al. An unusual crystal structure of ferric-enterobactin bound FepB suggests novel functions of FepB in microbial iron uptake. *Biochem Biophys Res Commun* **478**, 1049-53 (2016).
5. Li, N. et al. Unique iron coordination in iron-chelating molecule vibriobactin helps *Vibrio cholerae* evade mammalian siderocalin-mediated immune response. *J Biol Chem* **287**, 8912-9 (2012).
6. Clarke, T.E., Braun, V., Winkelmann, G., Tari, L.W. & Vogel, H.J. X-ray crystallographic structures of the Escherichia coli periplasmic protein FhuD bound to hydroxamate-type siderophores and the antibiotic albomycin. *J Biol Chem* **277**, 13966-72 (2002).
7. Drage, M.G. et al. *Mycobacterium tuberculosis* lipoprotein LprG (Rv1411c) binds triacylated glycolipid agonists of Toll-like receptor 2. *Nat Struct Mol Biol* **17**, 1088-95 (2010).
8. Sulzenbacher, G. et al. LppX is a lipoprotein required for the translocation of phthiocerol dimycocerosates to the surface of *Mycobacterium tuberculosis*. *Embo J* **25**, 1436-44 (2006).
9. Zajonc, D.M. et al. Molecular mechanism of lipopeptide presentation by CD1a. *Immunity* **22**, 209-19 (2005).
10. Sambandamurthy, V.K. et al. *Mycobacterium tuberculosis* D*RD1* D*panCD*: a safe and limited replicating mutant strain that protects immunocompetent and immunocompromised mice against experimental tuberculosis. *Vaccine* **24**, 6309-20 (2006).
11. Jain, P. et al. Specialized transduction designed for precise high-throughput unmarked deletions in *Mycobacterium tuberculosis*. *MBio* **5**, e01245-14 (2014).
12. Wells, R.M. et al. Discovery of a siderophore export system essential for virulence of *Mycobacterium tuberculosis*. *PLoS Pathog* **9**, e1003120 (2013).
13. Jones, C.M. & Niederweis, M. *Mycobacterium tuberculosis* can utilize heme as an iron source. *J Bacteriol* **193**, 1767-70 (2011).
14. Snapper, S.B., Melton, R.E., Mustafa, S., Kieser, T. & Jacobs, W.R., Jr. Isolation and characterization of efficient plasmid transformation mutants of *Mycobacterium smegmatis*. *Mol Microbiol* **4**, 1911-9 (1990).
15. Ofer, N. et al. Ectoine biosynthesis in *Mycobacterium smegmatis*. *Appl Environ Microbiol* **78**, 7483-6 (2012).
16. Alland, D., Steyn, A.J., Weisbrod, T., Aldrich, K. & Jacobs, W.R., Jr. Characterization of the *Mycobacterium tuberculosis iniBAC* promoter, a promoter that responds to cell wall biosynthesis inhibition. *J. Bacteriol.* **182**, 1802-11 (2000).
