# Supplementary material for: A periplasmic cinched protein is required for siderophore secretion and virulence of Mycobacterium tuberculosis
Source: Nat Commun. 2022 Apr 26;13:2255. doi: 10.1038/s41467-022-29873-6 (PMC9042941; doi:10.1038/s41467-022-29873-6)
Supplement: Supplementary file 2 — Reporting Summary [file 41467_2022_29873_MOESM2_ESM.pdf]

nature portfolio

Corresponding author(s): Michael Niederweis

Last updated by author(s): Mar 27, 2022

Reporting Summary

Nature Portfolio wishes to improve the reproducibility of the work that we publish. This form provides structure for consistency and transparency in reporting. For further information on Nature Portfolio policies, see our Editorial Policies and the Editorial Policy Checklist.

Statistics

For all statistical analyses, confirm that the following items are present in the figure legend, table legend, main text, or Methods section.

n/a	Confirmed	
☐	☒	The exact sample size (*n*) for each experimental group/condition, given as a discrete number and unit of measurement
☐	☒	A statement on whether measurements were taken from distinct samples or whether the same sample was measured repeatedly
☐	☒	The statistical test(s) used AND whether they are one- or two-sided<br>*Only common tests should be described solely by name; describe more complex techniques in the Methods section.*
☐	☒	A description of all covariates tested
☐	☒	A description of any assumptions or corrections, such as tests of normality and adjustment for multiple comparisons
☐	☒	A full description of the statistical parameters including central tendency (e.g. means) or other basic estimates (e.g. regression coefficient) AND variation (e.g. standard deviation) or associated estimates of uncertainty (e.g. confidence intervals)
☐	☒	For null hypothesis testing, the test statistic (e.g. *F*, *t*, *r*) with confidence intervals, effect sizes, degrees of freedom and *P* value noted<br>*Give P values as exact values whenever suitable.*
☒	☐	For Bayesian analysis, information on the choice of priors and Markov chain Monte Carlo settings
☒	☐	For hierarchical and complex designs, identification of the appropriate level for tests and full reporting of outcomes
☒	☐	Estimates of effect sizes (e.g. Cohen's *d*, Pearson's *r*), indicating how they were calculated

Our web collection on statistics for biologists contains articles on many of the points above.

Software and code

Policy information about availability of computer code

Data collection	Western blots were imaged using a Li-Cor Odyssey.<br>Lipidomics was performed using an Agilent 6520 Accurate-Mass Quadrupole Time of Flight Mass Spectrometer with an electrospray source and an Agilent 1200 series HPLC system with a Poroshell C18 column (3 mm x 50 mm).<br>Plate reader data was collected using a Biotek Synergy HT plate reader.<br>NMR spectra was performed using a Bruker Avance II (700 MHz 1H) spectrometer equipped with a cryogenic triple-resonance probe.<br>X-ray data was collected at the Stanford Synchrotron Radiation Lightsource (SSRL) using beam line 9-2.
Data analysis	Raw data for experiments was analyzed using Microsoft Excel and Graphpad Prism v8.<br>Structural data was visualized and analyzed with PyMol 2.5.<br>All statistical analysis was performed using Graphpad Prism v8.<br>Quantitative image analysis of western blots was done using ImageJ 1.46r.

For manuscripts utilizing custom algorithms or software that are central to the research but not yet described in published literature, software must be made available to editors and reviewers. We strongly encourage code deposition in a community repository (e.g. GitHub). See the Nature Portfolio guidelines for submitting code & software for further information.

Data

Policy information about availability of data

All manuscripts must include a data availability statement. This statement should provide the following information, where applicable:

- Accession codes, unique identifiers, or web links for publicly available datasets
- A description of any restrictions on data availability
- For clinical datasets or third party data, please ensure that the statement adheres to our policy

The source data underlying figures is provided in an Excel file. Any other files or data are available upon request. The datasets generated during the crystallographic analysis of MSMEG_3494 protein (PDB ID: 7REF) is available at the Protein Data Bank at https://www.rcsb.org/.

Field-specific reporting

Please select the one below that is the best fit for your research. If you are not sure, read the appropriate sections before making your selection.

[x] Life sciences   [ ] Behavioural & social sciences   [ ] Ecological, evolutionary & environmental sciences

For a reference copy of the document with all sections, see nature.com/documents/nr-reporting-summary-flat.pdf

Life sciences study design

All studies must disclose on these points even when the disclosure is negative.

Sample size	For the infection experiments, four mice for each time point per Mtb strain were used to determine the bacterial burden. For growth assays and alamar blue assays, three biological replicates each strain are used to determine mean +/- SD. For lipidomics experiments, four biological replicates each strain are used to determine mean +/- SD with significant difference analysis with one-way ANOVA with Tukey's test.
Data exclusions	No data were excluded from the analysis.
Replication	Experiments were performed 2-3 times to ensure reproducibility of findings unless otherwise indicated. For experiments which were performed twice, the results were already corroborated through an alternative experiment and were performed in order to visualize the results using multiple experimental approaches. For western blot experiments, representative blots were selected from two successful independent experiments. All the other data were repeated at least twice independently with good reproducibility.
Randomization	For all growth experiments, lipidomics, protein expression, and mouse infection experiment, colonies for all strains were randomly selected from solid agar plates.
Blinding	Blinding was not relevant to data analysis and was not performed during collection of the data. In all experiments, data collection, analysis and validation were performed by the different experimenters who independently collected and analyzed the data corresponding to the experiments they performed.

Reporting for specific materials, systems and methods

We require information from authors about some types of materials, experimental systems and methods used in many studies. Here, indicate whether each material, system or method listed is relevant to your study. If you are not sure if a list item applies to your research, read the appropriate section before selecting a response.

Materials & experimental systems

n/a	Involved in the study	
[ ]	[x]	Antibodies
[x]	[ ]	Eukaryotic cell lines
[x]	[ ]	Palaeontology and archaeology
[ ]	[x]	Animals and other organisms
[x]	[ ]	Human research participants
[x]	[ ]	Clinical data
[x]	[ ]	Dual use research of concern

Methods

n/a	Involved in the study	
[x]	[ ]	ChIP-seq
[x]	[ ]	Flow cytometry
[x]	[ ]	MRI-based neuroimaging

Antibodies

Antibodies used	The antibodies used in this study are described in the Method and source data file with their information. The anti-Rv0455c antibody was self-made. The anti-EccB5 antibody was obtained from Dr. Wilbert Bitter. The anti-Ag85 (NR-13800) and anti-CFP10 (NR-13801) antibodies were obtained from BEI Resources. The LI-COR IRDye 680RD Donkey anti-Rabbit lgG (926-68073) was used as secondary antibody.

Validation	Raw blots are available in the source data file. The electrophoretic mobility of all proteins recognized by the antibodies used in this study were converted to apparent molecular weights using marker proteins. The specificity of antibodies targeting was verified by using controls in the study. Complete blots are available in the source data file. In these blots we used as validation the molecular weights for the EccB5, Ag85, and CFP10 proteins, which have been published by our laboratory and by others.

Animals and other organisms

Policy information about studies involving animals; ARRIVE guidelines recommended for reporting animal research

Laboratory animals	7-8 weeks old female C57BL/6 mice (Jackson Laboratory)
Wild animals	No wild animals were used in the study.
Field-collected samples	No field collected samples were used in the study.
Ethics oversight	All animal experiments were performed following National Institutes of Health guidelines for housing and care of laboratory animals and performed in accordance with institutional regulations after protocol review and approval by the Institutional Animal Care and Use Committee of Weill Cornell Medical College (Protocol Number 0601441A).

Note that full information on the approval of the study protocol must also be provided in the manuscript.
